# Supplementary material for: Allergenic Activity of Individual Cat Allergen Molecules
Source: Int J Mol Sci. 2023 Nov 24;24(23):16729. doi: 10.3390/ijms242316729 (PMC10706119; doi:10.3390/ijms242316729)
Supplement: Supplementary file 1 [file ijms-24-16729-s001.zip › Table S3 Trifonova IJMS.pdf]

Table S3. Average percentages of beta-hexosaminidase release after stimulation with 10ng/ml of each of the cat allergens in cat-sensitized subjects, non-allergic subjects and allergic subjects without cat allergy.

Cat-sensitized children							
	Fel d 1	Fel d 2	Fel d 3	Fel d 4	Fel d 6	Fel d 7	Fel d 8
#51	11	0	1	0	1	2	2
#10	6	2	0	2	0	6	1
#56	74	53	63	78	38	66	60
#4	46	0	0	0	0	0	0
#7	0	0	9	31	0	42	6
#29	70	66	20	65	16	69	28
#23	71	0	0	9	2	4	3
#54	28	1	1	26	4	5	1
#5	34	0	0	17	0	0	0
#24	46	0	0	0	0	9	0
#13	61	0	2	11	0	0	1
#36	63	0	0	2	2	39	1.6
#27	67	45	47	59	9	38	25
#26	81	0	0	0	0	63	0
#34	17	0	0	0	0	12	0
#12	54	2	0	2	1	0	3
#16	0	0	0	16	0	67	0

Non-allergic subjects							
	Fel d 1	Fel d 2	Fel d 3	Fel d 4	Fel d 6	Fel d 7	Fel d 8
NA1	0	0	0	0	0	0	0
NA2	0	0	0	0	0	0	0
NA3	0	0	0	0	0	0	0
NA4	0	0	0	0	0	0	0
NA5	0	0	0	0	0	0	0
NA6	0	2	4	0	0	0	0
NA7	0	0	0	0	0	0	0
NA8	0	0	0	0	0	0	0
NA9	0	0	0	0	0	0	0
NA10	0	0	0	0	0	0	0
NA11	0	0	0	0	0	0	0

Allergic subjects without cat allergy							
	Fel d 1	Fel d 2	Fel d 3	Fel d 4	Fel d 6	Fel d 7	Fel d 8
NA12	0	0	0	0	0	0	0
NA13	0	0	0	0	0	7	0
NA14	1	0	0	0	0	0	0
NA15	0	0	0	0	0	0	0
NA16	0	0	0	0	0	0	0
NA17	0	0	0	0	0	0	0

NA18	0	0	0	0	0	0	0
NA19	0	0	0	0	0	0	0
NA20	0	0	0	0	0	0	0
