# Supplementary material for: Allergenic Activity of Individual Cat Allergen Molecules
Source: Int J Mol Sci. 2023 Nov 24;24(23):16729. doi: 10.3390/ijms242316729 (PMC10706119; doi:10.3390/ijms242316729)
Supplement: Supplementary file 1 [file ijms-24-16729-s001.zip › Figure S1 Trifonova IJMS.pdf]

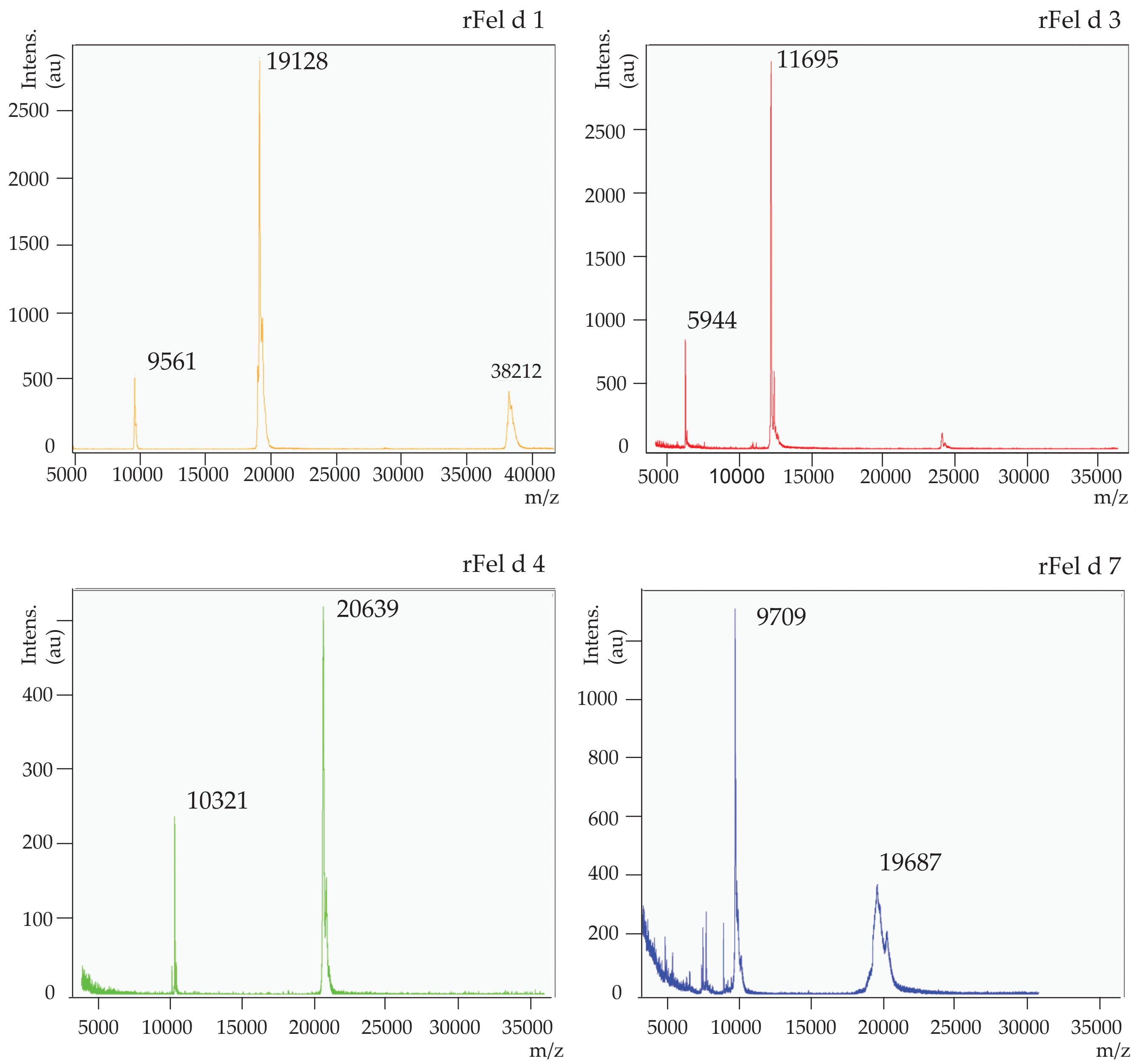

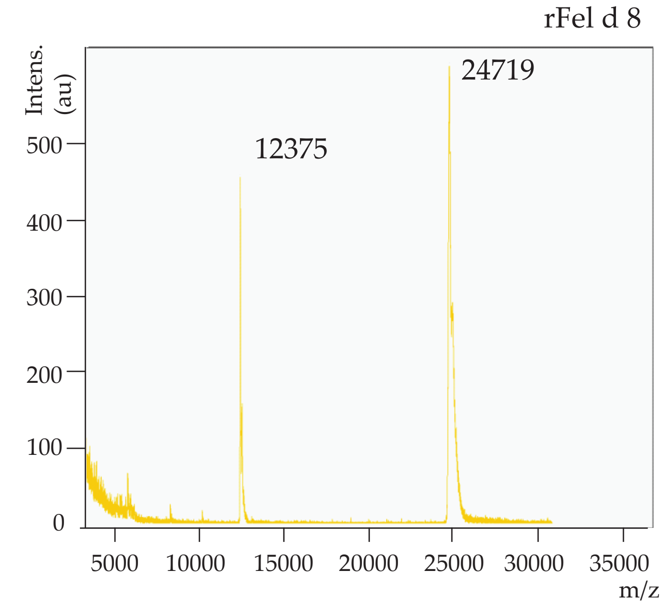

Figure S1. Mass spectrometry of cat allergens Fel d 1-8. Molecular mass was determined by matrix-assisted laser desorption/ionization time-of-flight mass spectrometry. Shown are signal intensities (y-axes) for the masses (x-axes).
