# Supplementary material for: Allergenic Activity of Individual Cat Allergen Molecules
Source: Int J Mol Sci. 2023 Nov 24;24(23):16729. doi: 10.3390/ijms242316729 (PMC10706119; doi:10.3390/ijms242316729)
Supplement: Supplementary file 1 [file ijms-24-16729-s001.zip › Table S1 Trifonova IJMS.pdf]

Table S1. Demographic, serological and clinical characteristics of cat-sensitized patients and their IgE reactivity profiles to individual cat allergen molecules

Patients			ImmunoCAP	Cat related symptoms					Other allergy				ImmunoCAP (kUA/L)								Sum Fel d		% of total						
#	Sex	Age	IgE (e1) kUA/L	respiratory	asthma	rhinitis	conj.	dermat.	dust	pet	pollen	food*	Fel d 1	Fel d 2	Fel d 3	Fel d 4	Fel d 6	Fel d 7	Fel d 8	**Sum**	1+4+7	Total IgE	Fel d 1	Fel d 2	Fel d 3	Fel d 4	Fel d 6	Fel d 7	Fel d 8
1	1	12	11.00	1	0	1	n.d.	n.d.	n.d.	1.2	1	1	13.30	0.00	0.76	2.50	0.00	0.79	1.06	18.41	16.59	324.44	4.10	0.00	0.23	0.77	0.00	0.24	0.33
2	1	10	0.39	0	0	0	n.d.	n.d.	n.d.	1.2	1	0	0.56	0.01	0.00	0.01	0.01	0.00	0.01	0.60	0.57	402.93	0.14	0.00	0.00	0.00	0.00	0.00	0.00
3	1	16	6.20	1	1	1	n.d.	n.d.	n.d.	1.2	1	1	7.70	0.04	0.07	0.08	0.04	0.24	0.04	8.21	8.02	593.26	1.30	0.01	0.01	0.01	0.01	0.04	0.01
4	2	16	7.40	1	0	1	n.d.	n.d.	n.d.	1	1	1	8.83	0.02	0.30	2.35	0.03	1.60	0.78	13.91	12.78	712.24	1.24	0.00	0.04	0.33	0.00	0.22	0.11
5	1	12	57.00	1	1	1	n.d.	n.d.	n.d.	1.2	1	1	72.10	0.04	1.36	12.40	0.10	0.03	3.17	89.20	84.53	946.99	7.61	0.00	0.14	1.31	0.01	0.00	0.33
6	1	14	0.19	0	0	0	n.d.	n.d.	n.d.	1	1	1	0.00	0.00	0.00	0.00	0.00	0.08	0.00	0.08	0.08	180.05	0.00	0.00	0.00	0.00	0.00	0.04	0.00
7	2	10	6.20	1	0	1	n.d.	n.d.	n.d.	1,2,3	1	1	0.01	0.78	1.48	17.70	0.04	22.20	2.97	45.18	39.91	528.58	0.00	0.15	0.28	3.35	0.01	4.20	0.56
8	1	12	0.76	0	0	0	n.d.	n.d.	n.d.	1	1	0	1.16	0.00	0.00	0.00	0.00	0.00	0.00	1.16	1.16	40.83	2.84	0.00	0.00	0.00	0.00	0.00	0.00
9	1	10	48.00	1	0	1	n.d.	n.d.	n.d.	1	1	0	59.10	0.01	0.01	0.01	0.02	0.01	0.01	59.17	59.12	417.35	14.16	0.00	0.00	0.00	0.00	0.00	0.00
10	1	15	2.70	1	1	1	n.d.	n.d.	n.d.	1	0	0	0.27	0.00	0.18	3.44	0.43	5.50	0.34	10.16	9.21	197.46	0.14	0.00	0.09	1.74	0.22	2.79	0.17
11	1	10	13.00	1	0	1	n.d.	n.d.	n.d.	1	1	1	21.50	0.06	0.05	0.32	0.05	0.56	0.09	22.63	22.38	777.48	2.77	0.01	0.01	0.04	0.01	0.07	0.01
12	1	15	9.50	0	0	0	n.d.	n.d.	n.d.	1.3	1	1	8.43	0.78	0.39	1.20	0.03	5.29	0.68	16.80	14.92	503.64	1.67	0.15	0.08	0.24	0.01	1.05	0.14
13	2	16	19.00	1	1	1	n.d.	n.d.	n.d.	1.2	1	1	24.00	0.05	1.75	4.01	0.07	0.03	2.73	32.64	28.04	730.35	3.29	0.01	0.24	0.55	0.01	0.00	0.37
14	2	14	2.10	1	1	0	n.d.	n.d.	n.d.	1	1	1	0.33	0.23	0.23	0.39	0.04	0.03	0.33	1.58	0.75	1354.60	0.02	0.02	0.02	0.03	0.00	0.00	0.02
15	1	12	2.80	0	0	0	n.d.	n.d.	n.d.	1	1	0	0.03	2.45	1.10	8.04	0.04	0.35	2.14	14.15	8.42	520.79	0.01	0.47	0.21	1.54	0.01	0.07	0.41
16	1	10	11.00	0	0	0	n.d.	n.d.	n.d.	1	1	1	0.09	0.05	0.39	12.90	0.05	52.50	0.94	66.92	65.49	898.63	0.01	0.01	0.04	1.44	0.01	5.84	0.10
17	2	16	4.40	1	1	1	n.d.	n.d.	n.d.	1	1	0	8.82	0.00	0.00	0.01	0.00	0.00	0.00	8.83	8.83	197.87	4.46	0.00	0.00	0.01	0.00	0.00	0.00
18	1	15	2.20	0	0	0	n.d.	n.d.	n.d.	1.2	1	1	2.66	0.00	0.08	0.26	0.00	0.11	0.12	3.23	3.03	181.06	1.47	0.00	0.04	0.14	0.00	0.06	0.07
19	2	14	7.20	0	0	0	n.d.	n.d.	n.d.	1.2	0	0	0.02	0.00	4.12	9.52	0.00	1.41	6.48	21.55	10.95	257.34	0.01	0.00	1.60	3.70	0.00	0.55	2.52
20	2	10	0.67	0	0	0	n.d.	n.d.	n.d.	1	0	0	0.00	0.00	0.00	0.00	0.00	0.00	0.00	0.00	0.00	262.15	0.00	0.00	0.00	0.00	0.00	0.00	0.00
21	1	10	1.40	1	0	1	n.d.	n.d.	n.d.	0	1	0	2.75	0.03	0.02	0.03	0.16	0.02	0.04	3.05	2.80	781.19	0.35	0.00	0.00	0.00	0.02	0.00	0.01
22	1	12	11.00	1	0	1	n.d.	n.d.	n.d.	1.2	1	1	26.00	0.32	0.07	0.51	0.01	0.50	0.17	27.58	27.01	414.42	6.27	0.08	0.02	0.12	0.00	0.12	0.04
23	1	12	47.00	0	0	0	n.d.	n.d.	n.d.	0	0	0	57.10	0.00	2.09	6.36	0.01	0.00	4.08	69.64	63.46	144.23	39.59	0.00	1.45	4.41	0.01	0.00	2.83
24	2	11	120.00	1	1	1	n.d.	n.d.	n.d.	1.2	0	0	136.00	0.00	0.02	0.01	0.01	5.54	0.01	141.59	141.55	250.35	54.32	0.00	0.01	0.00	0.00	2.21	0.00
25	1	11	7.70	1	0	1	n.d.	n.d.	n.d.	1.3	1	1	13.20	0.04	0.06	1.34	0.03	2.98	0.18	17.83	17.52	755.63	1.75	0.01	0.01	0.18	0.00	0.39	0.02
26	2	12	39.00	1	0	1	n.d.	n.d.	n.d.	1,2,3	1	1	48.30	0.03	0.87	1.45	0.05	22.30	1.52	74.52	72.05	502.10	9.62	0.01	0.17	0.29	0.01	4.44	0.30
27	1	11	840.00	1	0	1	n.d.	n.d.	n.d.	1.2	1	1	751.00	45.80	30.00	314.00	28.40	546.00	54.88	1770.08	1611.00	2698.17	27.83	1.70	1.11	11.64	1.05	20.24	2.03
28	1	15	12.00	1	0	1	n.d.	n.d.	n.d.	1	1	1	17.70	0.00	0.01	0.23	0.00	0.00	0.03	17.97	17.93	168.84	10.48	0.00	0.01	0.14	0.00	0.00	0.02
29	1	15	590.00	1	0	1	n.d.	n.d.	n.d.	1.2	1	0	342.00	446.00	9.35	67.50	30.40	83.80	16.90	995.95	493.30	1365.01	25.05	32.67	0.68	4.95	2.23	6.14	1.24
30	2	13	12.00	0	0	0	n.d.	n.d.	n.d.	1.2	1	1	15.50	0.09	0.09	0.45	0.11	0.32	0.12	16.68	16.27	2482.53	0.62	0.00	0.00	0.02	0.00	0.01	0.00
31	2	16	0.63	0	0	0	n.d.	n.d.	n.d.	0	1	1	1.03	0.11	0.11	0.11	0.13	0.07	0.14	1.70	1.21	3592.90	0.03	0.00	0.00	0.00	0.00	0.00	0.00
32	2	11	52.00	0	0	0	n.d.	n.d.	n.d.	1	1	0	101.50	0.01	0.00	0.01	0.02	4.78	0.01	106.33	106.29	360.47	28.16	0.00	0.00	0.00	0.01	1.33	0.00
33	2	15	5.60	1	0	1	n.d.	n.d.	n.d.	1.2	1	0	7.27	0.00	0.00	0.00	0.00	0.12	0.00	7.39	7.39	46.89	15.50	0.00	0.00	0.00	0.00	0.26	0.00
34	1	13	49.00	1	0	1	n.d.	n.d.	n.d.	2	1	0	81.20	0.04	0.03	0.17	0.03	10.70	0.06	92.23	92.07	778.28	10.43	0.01	0.00	0.02	0.00	1.37	0.01
35	1	16	7.70	1	1	1	n.d.	n.d.	n.d.	1,2,3	1	0	10.90	0.02	0.02	0.02	0.10	0.94	0.02	12.02	11.86	282.48	3.86	0.01	0.01	0.01	0.04	0.33	0.01
36	2	14	87.00	1	1	1	n.d.	n.d.	n.d.	1,2,3	1	0	205.00	0.37	0.40	11.00	0.32	63.40	1.15	281.64	279.40	7450.00	2.75	0.00	0.01	0.15	0.00	0.85	0.02
37	1	15	26.00	1	0	1	n.d.	n.d.	n.d.	2	1	1	48.90	0.01	0.01	0.01	0.02	0.00	0.01	48.96	48.91	423.50	11.55	0.00	0.00	0.00	0.00	0.00	0.00
38	1	14	180.00	1	0	1	n.d.	n.d.	n.d.	1.2	1	0	224.00	0.21	0.15	0.16	0.23	0.39	0.18	225.32	224.55	1639.77	13.66	0.01	0.01	0.01	0.01	0.02	0.01
39	1	17	4.80	1	1	1	n.d.	n.d.	n.d.	1.2	1	0	9.44	0.00	0.00	0.55	0.01	0.00	0.02	10.02	9.99	277.07	3.41	0.00	0.00	0.20	0.00	0.00	0.01
40	2	16	0.19	0	0	0	n.d.	n.d.	n.d.	2	0	1	0.01	0.01	0.01	0.03	0.00	0.17	0.01	0.24	0.21	393.59	0.00	0.00	0.00	0.01	0.00	0.04	0.00
41	1	13	200.00	1	1	1	n.d.	n.d.	n.d.	1.3	0	0	249.00	0.02	0.00	0.08	0.04	0.02	0.03	249.19	249.10	600.00	41.50	0.00	0.00	0.01	0.01	0.00	0.01
42	2	16	5.00	0	0	0	n.d.	n.d.	n.d.	1	0	1	5.51	0.02	0.00	0.03	0.04	0.65	0.04	6.29	6.19	788.75	0.70	0.00	0.00	0.00	0.01	0.08	0.01
43	1	17	0.49	0	0	0	n.d.	n.d.	n.d.	1	1	1	0.99	0.00	0.00	0.00	0.01	0.00	0.02	1.02	0.99	165.53	0.60	0.00	0.00	0.00	0.01	0.00	0.01
44	2	14	15.00	1	0	1	n.d.	n.d.	n.d.	1.3	1	1	17.30	0.05	0.00	3.24	0.09	0.15	0.27	21.10	20.69	1578.03	1.10	0.00	0.00	0.21	0.01	0.01	0.02
45	1	11	2.40	1	1	1	n.d.	n.d.	n.d.	1,2,3	0	1	2.77	0.15	0.48	0.75	0.00	2.06	0.71	6.92	5.58	121.03	2.29	0.12	0.40	0.62	0.00	1.70	0.59
46	1	10	6.50	1	1	1	n.d.	n.d.	n.d.	1.2	1	0	13.10	0.00	0.00	0.01	0.00	0.00	0.01	13.12	13.11	189.29	6.92	0.00	0.00	0.01	0.00	0.00	0.01
47	1	17	0.22	1	0	1	n.d.	n.d.	n.d.	0	1	0	0.44	0.00	0.00	0.00	0.00	0.00	0.01	0.45	0.44	95.83	0.46	0.00	0.00	0.00	0.00	0.00	0.01
48	1	10	0.27	0	0	0	n.d.	n.d.	n.d.	2	0	0	0.00	0.00	0.04	1.89	0.00	0.31	0.09	2.33	2.20	93.63	0.00	0.00	0.04	2.02	0.00	0.33	0.10
49	2	12	17.00	1	1	1	n.d.	n.d.	n.d.	1.2	1	1	25.50	0.04	0.14	0.78	0.15	1.33	0.17	28.11	27.61	1657.27	1.54	0.00	0.01	0.05	0.01	0.08	0.01
50	1	11	72.00	0	0	0	n.d.	n.d.	n.d.	1.3	1	1	113.56	0.06	0.15	0.16	0.16	2.43	0.15	116.67	116.15	1169.29	9.71	0.01	0.01	0.01	0.01	0.21	0.01
51	1	12	2.50	1	0	1	n.d.	n.d.	n.d.	1,2,3	0	0	4.99	0.25	0.24	0.44	0.46	1.06	0.33	7.77	6.49	2592.49	0.19	0.01	0.01	0.02	0.02	0.04	0.01
52	2	16	12.00	1	1	1	n.d.	n.d.	n.d.	0	0	1	8.37	0.03	6.44	25.50	0.03	4.03	10.00	54.40	37.90	781.74	1.07	0.00	0.82	3.26	0.00	0.52	1.28
53	1	12	0.37	0	0	0	n.d.	n.d.	n.d.	0	1	1	0.00	0.00	0.00	0.01	0.00	0.00	0.01	0.02	0.01	256.94	0.00	0.00	0.00	0.00	0.00	0.00	0.00
54	1	15	24.00	1	0	1	n.d.	n.d.	n.d.	1.2	1	1	52.70	0.13	0.28	0.56	0.23	10.80	0.40	65.10	64.06	3004.00	1.75	0.00	0.01	0.02	0.01	0.36	0.01
55	2	10	22.00	1	1	0	n.d.	n.d.	n.d.	1,2,3	1	1	29.90	0.01	0.01	0.16	0.01	4.54	0.03	34.66	34.60	269.69	11.09	0.00	0.00	0.06	0.00	1.68	0.01
56	1	10	350.00	1	1	1	n.d.	n.d.	n.d.	1.2	1	1	258.00	12.80	21.80	44.60	0.46	45.80	35.10	418.56	348.40	1369.42	18.84	0.93	1.59	3.26	0.03	3.34	2.56
57	2	12	4.40	0	0	0	n.d.	n.d.	n.d.	1.2	0	1	0.70	1.26	0.01	0.02	0.02	0.00	0.01	2.02	0.72	726.87	0.10	0.17	0.00	0.00	0.00	0.00	0.00
	**Male to female ratio: 36/21**	**Mean (min-max)**		**Sum (N of positives)**									**Mean for positive results (min-max)**																
		**13.1 (10-17)**	**53.3 (0.19 - 840)**	**37**	**17**	**35**				**51**	**44**	**33**	**64.8 (0-751)**	**31.9 (0-446)**	**3.4 (0-30)**	**15.4 (0-314)**	**4.1 (0-30.4)**	**25.15 (0-546)**	**4.9 (0-54.9)**	**99.6 (0-1770)**	**80.24 (0-1611)**	**882.37 (49.83-3592.9)**							

Symptomes:	1 Yes
	0 No

1	dog
2	horse
3	other furry animal
0	no allergy

*Food allergy: class 1 and class 2 food allergens
