# Supplementary material for: Allergenic Activity of Individual Cat Allergen Molecules
Source: Int J Mol Sci. 2023 Nov 24;24(23):16729. doi: 10.3390/ijms242316729 (PMC10706119; doi:10.3390/ijms242316729)
Supplement: Supplementary file 1 [file ijms-24-16729-s001.zip › Table S2 Trifonova IJMS.pdf]

Table S2. Demographic and clinical characteristics of subjects from control groups.

Non-allergic subjects

			Other allergy				
#	Sex	Age	HDM	Pet	Tree pollen	Grass pollen	Mold/Latex
1	m	25	no	no	no	no	no
2	m	35	no	no	no	no	no
3	m	30	no	no	no	no	no
4	m	42	no	no	no	no	no
5	m	26	no	no	no	no	no
6	m	35	no	no	no	no	no
7	f	30	no	no	no	no	no
8	m	40	no	no	no	no	no
9	f	26	no	no	no	no	no
10	m	30	no	no	no	no	no
11	f	42	no	no	no	no	no
	**Male to female ratio: 8/3**	**Mean (min-max): 32.8 (25-42)**	**0**	**0**	**0**	**0**	**0**

Allergic patients without cat allergy

			Other allergy				
#	Sex	Age	HDM	Pet	Tree pollen	Grass pollen	Mold/Latex
12	m	40	yes	no	no	yes	no
13	f	42	yes	no	yes	no	no
14	f	39	no	no	no	yes	no
15	m	25	no	no	yes	yes	no
16	m	22	no	no	yes	yes	yes
17	f	33	yes	no	no	no	no
18	f	22	no	no	yes	yes	no
19	m	30	yes	no	no	no	no
20	m	18	no	no	no	yes	yes
	**Male to female ratio: 4/5**	**Mean (min-max): 30.1 (18-42)**	**4**	**0**	**4**	**6**	**2**
